# The Unique Carboxymethyl Fenugreek Gum Gel Loaded Itraconazole Self-Emulsifying Nanovesicles for Topical Onychomycosis Treatment

**DOI:** 10.3390/polym14020325

**Published:** 2022-01-14

**Authors:** Ali Alqahtani, Bhavana Raut, Shagufta Khan, Jamal Moideen Muthu Mohamed, Adel Al Fatease, Taha Alqahtani, Ali Alamri, Fazil Ahmad, Venkatesan Krishnaraju

**Affiliations:** 1 Department of Pharmacology, College of Pharmacy, King Khalid University, Guraiger, Abha 62529, Saudi Arabia; amsfr@kku.edu.sa (A.A.); ttaha@kku.edu.sa (T.A.); krishcology@gmail.com (V.K.); 2 Institute of Pharmaceutical Education and Research, Borgaon (Meghe) Wardha, Wardha 442001, India; bhavanaraut23@gmail.com; 3 College of Pharmacy, Shri Indra Ganesan Institute of Medical Science, Tiruchirapalli 620012, India; jmuthumohamed@gmail.com; 4 Department of Pharmaceutics, College of Pharmacy, King Khalid University, Guraiger, Abha 62529, Saudi Arabia; afatease@kku.edu.sa (A.A.F.); aamri@kku.edu.sa (A.A.); 5 Department of Anesthesia Technology, College of Applied Medical Sciences in Jubail, Imam Abdulrahman Bin Faisal University, Dammam 34212, Saudi Arabia; fmahmad@iau.edu.sa

**Keywords:** itraconazole, onychomycosis, self-emulsifying nanovesicles, transungual, anti-fungal

## Abstract

The novel itraconazole (ITZ) nail penetration enhancing self-emulsifying nanovesicles (ITZ-nPEVs) loaded in carboxymethyl fenugreek gum (CMFG) gel circumvent the systemic onychomycosis treatment. The ITZ-nPEVs were prepared by the thin film hydration technique, and the particle size (PS), zeta potential (ZP), drug content (DC), entrapment efficiency (% EE), deformity index (DI), viscosity, morphology, and physical stability of the ITZ-nPEVs were measured. In terms of nail hydration, transungual drug absorption, and antifungal efficacy against *Candida albicans*, the chosen ITZ-nPEVs, nPEV-loaded CMFG (CMFG-ITZ-nPEVs) gel, and the commercialized Itrostred gel were compared. The ITZ-nPEVs showed spherical structure with high DC, % EE, low PS and PDI and positive ZP of ITZ ranging from 95.36 to 93.89 mg/5 mL and 95.36–96.94%, 196.55–252.5 nm, 0.092–0.49, and +11.1 to +22.5 mV, respectively. Compared to the Itrostred gel, the novel ITZ-nPEVs exhibited hydration enhancement factor for 24 h (HE24) of 1.53 and 1.39 drug uptake enhancement factor into nail clippings. Moreover, zone of inhibitions for ITZ-nPEVs (27.0 ± 0.25 mm) and CMFG-ITZ-nPEVs (33.2 ± 0.09 mm) against *Candida albicans* were significantly greater than that of Itrostred gel (22.9 ± 0.44 mm). For clinical investigation on onychomycotic patients, a nail penetration enhancer containing ITZ-nPEV-loaded CMFG gel presents a highly promising approach.

## 1. Introduction

Onychomycosis is a fungal infection that affects both the fingernail and toenail. The documented negative effects of antifungal medication, as well as the restricted blood circulation to the afflicted nails, have impeded systemic therapy of onychomycosis. Approximately 19% of the global population is affected by the fungal infection of the human nail, which is known as onychomycosis or tinea unguium [1]. *Trichophyton rubrum*, followed by *Trichophyton mentagrophtes* var; interdigitale, are the anthropophilic dermatophytes that cause this illness. Non-dermatophytes molds, such as *Scopulariopsis brevicaulis* and *Aspergillus* spp., can be main and secondary pathogens in onychomycosis. Yeast, like *Candida albicans* and *Candida parapsilosis*, is the third cause of nail fungal infection [2].

Onychomycosis causes thickening and discoloration of nail. The nail becomes brittle and begins to break or completely come out of the toe or finger as the infection develops [3]. The toenail is determined to be the most impacted by the fungal infection of all the nails, whereas fingernails are the least affected [4]. Because of the low vascularity of the nail bed and barrier characteristic of the nail plate, penetration of drugs through the nail is very poor, the human nail is composed of 25 keratinized layers, which is 100-fold thicker than the stratum corneum. Current treatment strategies include both oral and topical delivery, but both suffer from poor diffusion of drugs through the nail [5]. Therefore, it is highly desirable to design formulations that can improve nail penetration of the antifungal drugs. To improve the therapeutic efficiency by the topical route, three key means include mechanical, physical and chemical. The mechanical therapy involves complete nail avulsion or filing the affected nail, the physical means include iontophoresis, phonophoresis, photodynamic therapy or laser therapy and chemical method uses chemical nail penetration enhancer [6]. Nanoparticles offer deep penetration of drug into the nail with prolonged retention in the nail and can avoid the painful surgical removal of the nail. Wang et al. (2018) demonstrated improved permeation of ketoconazole through the nail plate and longer its retention at the site when it was encapsulated in crosslinked fluorescent supramolecular nanoparticles [7]. Nail-penetrating nanovesicles have shown promise in improving diffusion of drugs through. Elsherif et al. (2018) formulated terbinafine hydrochloride-loaded spanlastic nanovesicular carrier for enhanced transungual drug delivery. The nanovesicles however have poor retention at the site. To improve both penetration and retention, nail penetration nanovesicles were dispersed in gel in the present investigation [8]. Itraconazole (ITZ) was used as a model drug and an attempt has been made to improve its solubility in the aqueous medium and improve its penetration.

The aim of research work is to develop an effective topical delivery system suitable for treatment of onychomycosis, to eliminate the need for systemic intervention. The nail penetration-enhancing vesicles open a new approach for topical treatment of nail-related fungal infection such as onychomycosis. In the present work for the formulation of nanovesicles, incorporation of the penetration enhancer labrasol, with the nail penetration enhancer N-acetyl-L-cysteine and the positive charge inducer stearylamine within aqueous deformable-natured nanovesicles (nPEVs) proved to be a promising combination for enhancing the transungual delivery of ITZ.

## 2. Results and Discussion

### 2.1. High-Performance Liquid Chromatography (HPLC)

Figure S1 shows the quantification and standard calibration curve for ITZ using the HPLC technique. With a correlation value of 0.9991, a linear response was seen in range of 5 to 50 µg/mL (Figure S1b and Table S1). The derivatized ITZ had a retention time of 7.8 min, a limit of identification of 110 µg/mL, and a limit of detection of 32 µg/mL (Figure S1a), respectively.

### 2.2. Preparation of ITZ-nPEVs

According to the earlier work [9], the ITZ-nPEVs were effectively produced by employing the thin film hydration approach. Briefly, amount of nail penetration enhancers was restricted to a maximum of 5% as greater concentrations were observed to soften the nail to clinically unacceptable levels (Table 1). Labrasol and N-acetyl-L-cysteine were used as enhancers in the synthesis of nPEVs. The nail penetration enhancer (N-acetyl-L-cysteine) was found to increase flux across the nail plate by diminishing disulfide linkages in the keratin of the nail, which was linked to pore formation and subsequent swelling and softening of the nail plate, resulting in a reduction in nail barrier integrity [10]. Because the nails are negatively charged at pH 7.4, the positive charge inducer stearylamine was added to help with transungual penetration [11]. Labrasol increases fluidity of the vesicles allowing greater deformability leading to greater penetration of vesicles through the pores [12] (Drug Delivery, 2017, 24(1), 98–108).

### 2.3. Drug Content and % EE

The drug content and % EE of ITZ-nPEVs ranged from 95.36 ± 0.517 to 193.89 ± 0.83 mg/5 mL of nPEVs and 95.36 ± 0.517 and 96.94 ± 0.70%, respectively (Table 1). These high % EE values can be ascribed to ITZ’s lipophilicity (log P = 5.66), which allows it to be effectively incorporated into lipid bilayers in the various formulations [13].

The drug content of ITZ-nPEVs of selected batch S3 was found to be 98.43 ± 0.32 mg in 5 mL of nPEVs and the % EE of selected batch S3 was found to be 97.22 ± 0.46%. It was observed that the selected batch S4 showed the maximum drug content and % EE due to increase in lipid and cholesterol ratio (2:7:3).

### 2.4. PS, PDI and ZP

The average particle size of ITZ-nPEVs varied from 196.55 ± 0.025 to 252.2 ± 0.019 nm, as reported in Table 1 and Figure S2a. The PDI values of ITZ-nPEVs did not exceed 0.4, indicating that the solution was homogeneous and monodisperse [14]. It was observed that the concentration of lipid increases, the particle size of formulated ITZ-nPEVs also increased. The particle size of S3 was found to be 240.33 ± 0.016 nm. Because of the presence of the positive charge inducer stearylamine, the zeta potential values varied from +11.1 to +22.5 mV (Figure S2b). The zeta potential of chosen batch S3 was found to be, 19.1 mV. According to Mohammed et al. (2021), the large magnitude of charge indicates good stability against vesicle aggregation and fusion [15].

The tiny particle size produced for all of the developed ITZ-nPEVs formulations (196.55 to 252.2 nm) is evident from Table 1. ITZ-nPEVs vesicles of this size have a significant interfacial surface area, which aids drug absorption and lymphatic transit [16]. For nanoformulations, relatively high polydispersity indices (>0.5) are considered typical. This is because the surfactant monolayer’s interfacial tension is very low for nanostructures, so there is less of a penalty (more chance) for having a non-spherical shape, compared to normal emulsions, which typically have spherical structures due to high interfacial tensions favoring globule interfacial areas reduction (The sphere has the lowest interfacial area for a given volume.).

### 2.5. Elasticity

The deformability index of S1- S4 ITZ-nPEVs were ranging from 1.35 to 0.200 mL·s^−1^ as shown in Table 2. The deformability index of selected batch S3 was found to be 0.449 mL·s^−1^. The S3 vesicles had a lower deformability index than S1 and S2 vesicles, which might be due to the lipid’s lesser ability to interact with the penetration enhancer when compared to cholesterol. The deformability of nanovesicles was owing to the presence of labrasol within the membrane vesicles, which confers fluidity, flexibility, and the ability to create vesicles so that they can deform, according to Yusuf et al. (2014) [17]. However, the phenomenon is relevant up to a particular surfactant concentration limit, after which mixed vesicles develop, which are hard vesicles with little or no deformability.

### 2.6. Viscosity

As shown in Table 2, the viscosity of ITZ-nPEVs and CMFG-ITZ-nPEVs ranged from 0.98 ± 0.02 to 2.41 ± 0.131 cP. Because of the presence of vesicular lamellar structures with a large hydrodynamic volume, the ITZ-nPEVs dispersions had greater viscosity values than water [4]. S4 ITZ-nPEVs had a much greater viscosity (1.72 cP) than CMFG-ITZ-nPEVs (2.41 ± 0.131 cP), which was significantly less.

At increasing shear rates, however, the viscosity steadily rises, indicating shear thickening behavior. Because the creation of the interparticle structure was hampered by electrostatic repulsion at low shear rates, the viscosity was Newtonian. Almahfood and Bai (2021) explained that the shear rate was greater than 120 s^−1^; however, the attraction of nanogel dispersions increased, causing the viscosity to steadily rise. Furthermore, when the shear rate increases, nanogel dispersions show an abrupt increase in viscosity values, which might be attributed to enhanced particle contact produced by the high rotating speed. Despite this, a larger concentration of nanogel dispersion did not follow the same pattern [18]. This might be explained by the microstructure of nanogel dispersions changing at greater shear rates.

### 2.7. ITZ- nPEVs Shape

**Figure 1:**
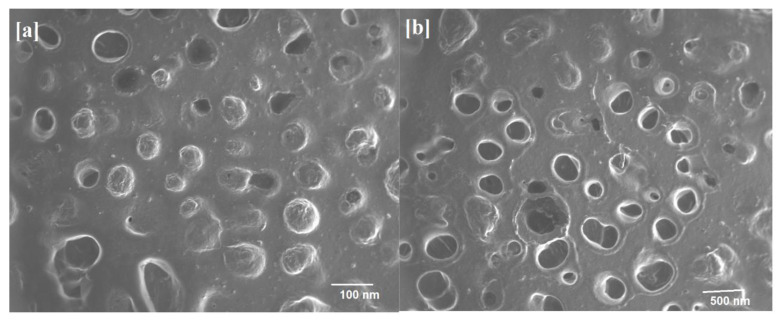
(**a**) SEM photograph of selected S3 and (**b**) size-measured vesicles.

The scanning electron microscopic (SEM) study was carried out on a selected batch (S3) of ITZ- nPEVs with drug, lipid, and surfactant ratio of 1:7:3 as shown in Figure 1. The border and core of well-identified vesicular structures with spherical shape may be seen in SEM of the nPEVs (Figure 1a). The structural appearance revealed a lighter core encompassed by a denser border that perfectly enclosed the center. When a thin lipid layer is hydrated, it develops enclosed vesicular network that supports in shape from spherical to circular in order to achieve thermodynamic stability by lowering the systems total free energy [19]. Even after applying various mechanical loads such as sonication and extrusion, no disturbances in vesicular structure proved vesicle integrity (Figure 1b).

### 2.8. In Vitro ITZ Release

**Figure 2:**
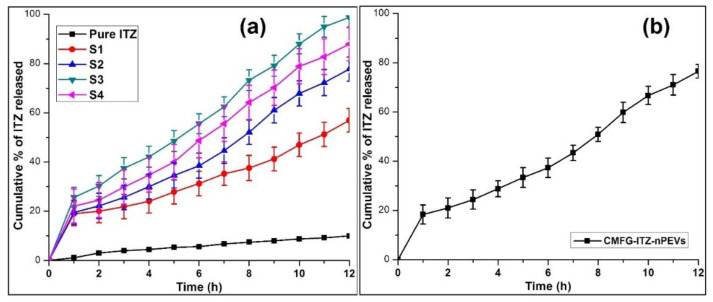
In vitro release of ITZ from (**a**) pure drug, S1-S4 ITZ-nPEVs, and (**b**) CMFG-ITZ-nPEVs (Mean ± SD).

The ITZ release of each batch and CMFG-ITZ-nPEVs was carried out by using dialysis membrane into the USP dissolution apparatus (Type II) for 12 h in phosphate buffer (pH 7.4). The cumulative ITZ release of all batches was in the range of 56.95 ± 0.21–98.75 ± 0.28 as shown in Figure 2a. The drug release of a selected batch S3 and CMFG-ITZ-nPEVs was found to be 98.75 ± 0.28% and 76.56 ± 2.77% for 12 h, as shown Figure 2b. The CMFG-ITZ-nPEVs have the ability to release the drug in controlled way, which is evident in the present investigation. Thus, constant/unhindered drug release over prolonged time could be achieved due to improvement in solubility of ITZ [17]. The slow ITZ release in case of free drug was because of its inherent poor aqueous solubility. ITZ belongs to the BCS II class [20].

Table 3 shows that the in vitro drug release was best described by Higuchi equation with the highest linearity (R^2^ = 0.9789) for optimized batch S3. Slope of Korsemayer–Peppas equation greater than 0.5 and less than 0.85 which indicates non-Fickian diffusion, i.e., drug release occurred by both diffusion and erosion [21].

### 2.9. Nail Hydration/Transungual Drug Uptake of ITZ-nPEVs 

The nail hydration average weight gain group 2 (S1–S4 batches) was found to be 62.0, 68.2, 75.6, and 72.7 mg, respectively. For, the groups I (control) and group 4 (Itrostred gel) were 49.2 and 52.65 mg, respectively. For chosen batch S3 and the marketed gel, the hydration enhancement factor HE24 values were 1.53 and 1.07, respectively (Table 4). The hydrophilic nature of formula S3 is likely to be responsible for the much larger weight gain seen when compared to Itrostred gel. In this instance, this was advantageous because water was considered to be the greatest nail plasticizer, resulting in greater drug flux across the nails [22].

The transungual uptake of ITZ was due to the effective partitioning of the drug into the nail clipping. The amount of ITZ taken up by the nail clippings exposed to S3 batch and Itrostred gel were 94.2% and 67.36%, respectively, the corresponding nail uptake enhancement factor EFnail for S3 batch was found to be 1.39 as compared to the marked gel [23].

**Figure 3:**
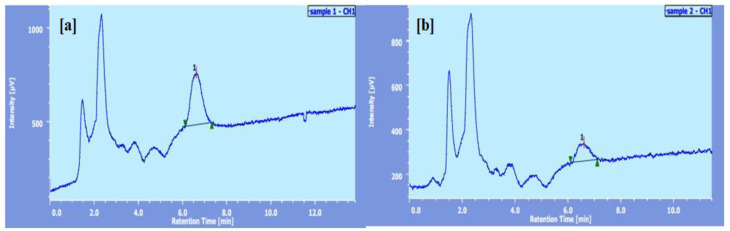
Chromatogram of (**a**) S3 batch and (**b**) Itrostred gel.

Figure 3 illustrates ITZ’s great affinity for nail clippings, as nPEVs allowed it to enter the nail in substantial numbers, allowing it to cure the deeply rooted onychomycosis infection.

### 2.10. The Efficacy of ITZ-nPEVs for the Treatment of Onychomycosis 

Several researchers suggested that *Candida albicans* (MTCC No. 227) be used to evaluate in vitro antifungal activity as it is the most common dermatophyte that causes onychomycosis [3,24]. ITZ is a fungistatic antifungal medication with a broad spectrum of activity (Table 5).

The antifungal activity of the formulations, which included vesicular dispersion (S3), plain unmediated formula (control), ITZ-nPEVs loaded gel, and commercial product (Itrostred gel), was tested using the agar diffusion technique [25]. The “zones of inhibition” are the transparent rings that emerge around the dishes. The more efficient the formulation, the bigger the zone of inhibition. Surprisingly, against *Candida albicans*, the simple unmedicated formula (control) revealed a mean zone of inhibition (5.1 0 ± 0.12 mm). This might be explained by the fact that cysteine and its derivatives (N-acetyl-L-cysteine) have been found to have antifungal properties.

The mean zone of inhibition for Formula S3 was 27.0 ± 0.25 mm, while the mean zone of inhibition for CMFG-ITZ-nPEVs gel was 33.2 ± 0.09 mm, which was substantially bigger than the mean zone of inhibition for the commercial preparation Itrsostred gel (22.9 ± 0.44 mm). This might be due to the larger release and diffusion potential of formulation CMFG-ITZ-nPEVs gel, as well as the antifungal potential of N-acetyl-L-cysteine and CMFG gel compared to the commercial preparation, resulting in more partitioning of ITZ from the preparation [26]. The increase in the zone of inhibition with CMFG-ITZ-nPEVs gel compared to ITZ-nPEVs could be because of the inherent potent antifungal activity of fenugreek [27]. Note that vesicles have been successfully used in the topical treatment of onychomycosis employing transfersomal, liposomal, and ethosomal terbinafine [4,28]. The findings show that nail penetration enhancers with nanovesicles (nPEVs) are a potential ungual delivery mechanism that can be used in clinical trials on onychomycotic patients.

### 2.11. Stability Study

The CMFG-ITZ-nPEVs gel was kept for stability study and further characterization studies. From the results shown in Table 6, it was observed that CMFG-ITZ-nPEVs gel was stable for period of 6 months at 45 ± 0.5 °C and 60% ± 5% RH. Upon storage, only a slight increase in particle size and PDI was observed (349.33 ± 0.92 nm and 0.41, respectively). The drug content, % EE, zeta potential and in vitro drug release of ITZ after storage was the same as before storage 98.21 ± 0.12 mg/100 mg of drug in 5 mL of nPEVs, 98.21 ± 0.12%, 19.6 mV and 98.79 ± 0.44%, respectively. This indicates that CMFG-ITZ-nPEVs have high physical stability when stored at 4 °C [29].

## 3. Materials and Methods

### 3.1. Reagents

Itraconazole (ITZ) was supplied as a gift from Glenmark Pharmaceutical Ltd., Nashik, India. Lecithin USP-NF (LECIVA-S75) was supplied as gift from VAV Life Sciences Pvt. Ltd., Mumbai, India. Labrasol was kindly provided by Gattefosse Pvt. Ltd., Mumbai, India. Stearylamine and Sabouraud dextrose agar (SDA) supplied from HiMedia Laboratories Pvt. Ltd., Mumbai, India and N-acetyl-L-cysteine, Cholesterol, Mono chloroacetic acid, HPLC grade methanol and water were supplied from LOBA chemical, Pvt Ltd., Mumbai, India. Itrostred gel containing 1% ITZ was purchased from Nisha Medicals, Tiruchirappalli, Tamil Nadu, India manufactured by Leeford Healthcare Ltd. Thana, Solan, India.

### 3.2. HPLC Analysis

Determination of ITZ was accomplished using a validated HPLC method (Model No. LC-10AD, Shimadzu, Kyoto, Japan). The mobile phase was a mixture of methanol: water containing (75:25 *v*/*v*). The flow rate of mobile phase was 1 mL/min and the injection volume was 10 µL [30]. Samples were injected into a C18 column (Hypersil, 250 × 4.6 i.d., particle size 5 µm) and the column effluent was monitored at 262 nm.

### 3.3. Preparation of ITZ-nPEVs

The self-emulsifying nanovesicles ITZ-nPEVs were made using a thin-film hydration approach followed by sonication, and the composition of the variously synthesized ITZ-nPEVs is presented in Table 1. Penetration enhancers such as Labrasol (200 mg), N-acetyl-L- cysteine (250 mg), and Stearylamine (20 mg) were carefully weighed and dissolved in a chloroform: methanol combination (2:1; *v*/*v*) in all of the manufactured ITZ-nPEVs. Under decreased pressure at 40 °C and 150 rpm, the organic solvent mixture was evaporated (Rotary evaporator, Model No. SB-1000, Tokyo Rikakikai Co., Ltd., Bunkyo-Ku, Japan) to form a thin layer of dry lipid containing the medicine on the inner wall of the flask [31]. Through portion-wise addition, the dry lipid film was hydrated with 5 mL of phosphate buffer (pH 7.4). The dispersion was mechanically rotated for 30 min at 40 °C, then sonicated for 15 min at a frequency of 33 KHz to minimize the size of the vesicles and stored at 4 °C (Model No. 1.5 L 50, PCI analytics, Mumbai, India).

### 3.4. Elimination of Unentrapped ITZ from ITZ-nPEVs

The unentrapped ITZ was removed from the nPEVs using Bseiso et al. (2015) exhaustive dialysis method. Briefly, the ITZ-nPEVs were integrated into dialysis tubing (MW. Cut off 12,000–14,000) and dialyzed against 1 L of double distilled water (pH 7.04) at room temperature for 24 h. Preliminary dialysis experiments guided the selection of these parameters [32].

### 3.5. Preparation of ITZ Self- Emulsifying Nanovesicles Gel (CMFG-ITZ- nPEVs)

#### 3.5.1. Extraction and Purification of Fenugreek Gum (FG)

Fenugreek seeds were cleaned, washed, and air-dried and then soaked in water overnight and the seeds were boiled in hot water for 3 h at 80 °C to extract the gum and inactivate the enzymes, respectively. The solution was then allowed to cool to room temperature before being pressed through a cotton towel. With the addition of an equivalent volume of acetone, the crude gum was precipitated from the ensuing viscous solution. Gum was purified by washing it in ethanol and then in acetone [33]. The purified gum was dried overnight at 50–60 °C in an oven. The dried gum was pulverized and sieved through mesh #100 and stored at room temperature for further use.

#### 3.5.2. Synthesis of Carboxymethyl Fenugreek Gum (CMFG)

The 1:1 ratio of FG and sodium bicarbonate mixed well in a mortar followed by the addition of ethanol (<0.01%) for the surface treatment of gel. The gel transferred to a flask fitted with a thermometer and mechanically stirred for 30 min. The solid mono-chloroacetic acid (1%) was added to the above gel in the presence of temperature 75 °C with continuous stirring for another 3 h. The reaction mixture was immediately cooled and neutralized to pH 7 using dilute acetic acid [34]. The gel was washed twice with methanol: water (80:20) followed by methanol washing and dried at 50–60 °C in oven an overnight.

#### 3.5.3. Preparation of CMFG-ITZ-nPEVs Gel

In the dark, Chen and colleagues described the synthesis of nanogel using a simple diffusion and dialysis approach [35]. In a nutshell, 50–100 mg chitosan was dissolved in 5 mL acetic acid at pH 3.0, and 50–100 mg CMFG (kept overnight for hydration in purified water) was dissolved in 5 mL dimethylformamide (DMF) by vortex and sonication (Table 2). Dropwise addition of CMFG solution into the chitosan solution was done for 12 h at 30 rpm at room temperature, then dialyzed for 24 h with deionized water. The dialysis medium was refreshed at least five times after the free CMFG and DMF were completely removed from the solution. Finally, the solution was filtered through a 0.22 m syringe filter before being lyophilized (Christ, Alpha1-2 LD plus, Osterode am Harz, Germany) to obtain the CMFG-ITZ-nPEVs gel, which was kept at 4 °C for 24 h.

### 3.6. Characterization

#### 3.6.1. Drug Content (DC)

After disrupting the dialyzed nPEVs with methanol, the quantity of ITZ entrapped in them was determined. To create a transparent solution, an aliquot of nPEVs was combined with an appropriate proportion of methanol and then covered with a parafilm to prevent methanol evaporation [17]. After adequate dilution, the concentration of ITZ was detected spectrophotometrically (Model No. UV 2401(PC), S.220 V, Shimadzu Corporation, Kyoto, Japan) at 262.4 nm. At this wavelength, there was no interference from blank nPEVs.

#### 3.6.2. % EE

The entrapment efficiency was calculated according to the following equations-

% Entrapment efficiency = (Actual drug content in ITZ-nPEVs)/(Therotical drug content in ITZ-nPEVs) × 100(1)

#### 3.6.3. Deformability Index

Extrusion was used to determine the deformability index of ITZ-nPEVs utilising a locally produced and approved Sartorius stainless steel pressure filter holder. The vesicles were computed using the following equation after being extruded through a membrane filter with a pore size of 50 nm at a constant pressure of 0.17 MPa [36].

D = j/t [rv/rp]^2^ (2)

where

D is the deformability index (mL·s^−1^),
j is the amount of vesicular dispersion extruded in mL,
t is the time of extrusion in second,
rv is the size of vesicles after extrusion (nm), and
rp is the pore size of the filter (nm).

#### 3.6.4. Viscosity of nPEVs

The prepared ITZ-nPEVs and CMFG-ITZ-nPEVs gel kept overnight for hydration in milliQ water and viscosity of that preparations were determined using Brookfield viscometer (Model No. CAP2000+L, Brookfield Engineering Lab., Middleborough, MA, USA) at 100 rpm using spindle No. 1 at 37 ± 0.5 °C [4].

#### 3.6.5. DLS

The practical size (PS; nm), polydispersity index (PDI) and zeta potential (ZP) of ITZ-nPEVs and CMFG-ITZ-nPEVs gel formulations was assessed by laser Doppler anemometry in triplicate by dynamic light scattering using zeta sizer (Model No. ZS90, Malvern Instruments Ltd., Worcestershire, UK). Light scattering was monitored at 25 °C and 90° angle after appropriate dilution [37,38].

#### 3.6.6. Morphology

SEM analysis (Model No. S3700N, Hitachi, Japan) was carried out on the selected ITZ-nPEVs formula in order to characterize the shape and ultrastructure of the vesicles according to the previous method described by Moideen et al. (2020) [38].

#### 3.6.7. In Vitro Drug Release and Kinetics

The in vitro drug diffusion study of ITZ-nPEVs formulation was evaluated using USP dissolution apparatus (Type II). A dialysis membrane (average diameter; 15.9 mm, average flat width; 25.27 mm, Himedia^®^, Mumbai, India) was hydrated with the phosphate buffer pH 7.4 for 12 h. Volume of ITZ-nPEVs formulation equivalent to 100 mg ITZ was dispersed in 5 mL of phosphate buffer and then placed in the bag of activated membrane which was sealed from both the ends. The dialysis bag then ties to the middle of the shaft of the USP apparatus (DA-3, Veego Scientific Mevices, Mumbai, India) and paddle was put into the jar containing 300 mL dissolution medium. The temperature of the study was controlled at 37 ± 0.5 °C under stirring at the speed of 50 rpm. A one milliliter aliquot was withdrawn at fixed time intervals and immediately replaced with an equal volume of fresh buffer to maintain the sink condition [3]. All samples were analyzed at 261.80 nm by UV Spectrophotometry (UV 2401(PC), S.220 V, Shimadzu Corporation, Kyoto, Japan). The experiment was done in triplicate to assess the drug diffusion characteristics from nPEVs formulation. Drug release was compared with plain ITZ for which 100 mg of ITZ was suspended in the 5 mL of buffer and study was carried out similar to ITZ-nPEVs. Drug release kinetics was assumed to reflect different release mechanism of controlled release drug delivery systems. Therefore, five kinetics model were applied to analyze the in vitro data to find the best fitting equation according to our previous study.

#### 3.6.8. Nail Hydration Study

Nail clippings were collected with nail clippers from healthy human volunteers (males and females, 25–50 years old). The current investigation employed just the middle, index, and ring fingernails as an in vitro model for evaluating transungual administration [39]. Nail clippings were thoroughly cleaned by washing five times with distilled water, wiping with tissue paper, and drying at 37 °C for 24 h before being kept in airtight containers [40]. Fifty milligrams of nail clippings was inserted in separate glass vials for the nail hydration experiment. For this investigation, three groups were formed: group I (control group), in which the pre-weighed nail clippings were immersed in 1 mL deionized water (pH 7.04), group II (nPEVs formulation), group III (nPEVs CMFG gel) and group IV (marketed Itrostred gel), in which the nail clippings were immersed in marketed Itrostred gel, which is equivalent to the 1 mL of nPEVs. The nail clippings were reweighed after thorough tissue paper wiping to quantify weight growth after the glass vials were sealed and incubated at room temperature for 24 h [9]. The following calculation was used to compute the hydration enhancement factor (HE) after 24 h (HE24):

HE 24 = (Weight gain of nail clippings of groups II/III/IV)/(Weight gain of nail clippings of group I)(3)

#### 3.6.9. Transungual Drug Uptake Study

The S3 (1:7:3) was the optimized formulation of ITZ-nPEVs from the in vitro drug release and nail hydration study. Groups 2 (optimized batch S3) and 3 (Itrostred gel) nail clippings were rinsed three times with methanol to eliminate any residues of medication on the surface, then dissolved in 1 M sodium hydroxide (1 mL) with continual overnight stirring [41]. The solutions were filtered through a 0.22 m syringe filter after full digestion of the nail clippings, and an aliquot was collected and diluted with methanol before HPLC analysis. The enhancement factor (EF nail) was computed using the following equation to represent the improvement in ITZ penetration into nPEV nail clippings as compared to marketed gel (Itrostred gel)

EF nail = (Extracted drug percentage in nail clippings of group II/III)/(Extracted drug percentage in nail clippings of group IV)(4)

#### 3.6.10. Anti-Microbiological Efficacy

The antifungal activity of the control, a chosen batch of nPEVs formula (S3), nPEVs loaded into the gel formulation, and the commercial preparation (Itrostred gel) was tested against *Candida albicans* (MTCC No. 227). One milliliter of the fungal culture suspension was combined with 9.9 mL liquid broth (without agar) and inoculated for 24 h at 25 degrees Celsius in an incubator (Remi instruments cooling incubator, Mumbai, India). 1 mL of inoculated liquid broth containing fungal culture suspension was poured to the sterile petri dishes containing solidified agar growth medium, and the inoculum was dispersed equally across the solid agar surface by turning the plate clockwise and anticlockwise.

With the use of a sterile cork-borer, wells were formed in the centre of the plates, and each well (6 mm internal diameter) was accurately filled with either 0.1 mL of control, ITZ-nPEVs, CMGF-ITZ-nPEVs gel, or Itrostred gel corresponding to ITZ dosage in the 0.1 mL ITZ-nPEVs. The plates were then incubated in the incubator for three days at 25 °C to allow for fungal development [42]. The antifungal activity was determined by measuring fungal growth inhibition zones around the formulations. They were measured on mm scale, and a comprehensive antifungal analysis was performed in an aseptic environment.

#### 3.6.11. Stability Study

The physical and chemical stability study of an ideal formulation of CMFG-ITZ-nPEVs at 45 ± 0.5 °C and 60 ± 5% RH for 3 months in stability chamber (Model No. HTC-3003, Wadegati TM Labe Quip (P) Ltd., Andheri (E), Mumbai, India). At an interval of 1 month, CMFG-ITZ-nPEVs gels analyzed for physical changes, drug content, particle size, zeta potential and in-vitro drug release.

## 4. Conclusions

The combination of the penetration enhancer labrasol, the nail penetration enhancer N-acetyl-L-cysteine, and the positive charge inducer stearylamine in aqueous deformable-natured nanovesicles (nPEVs) was proven to be a viable combination for improving ITZ transungual administration. The prepared CMGF-ITZ-nPEVs gel satisfy best attributes for topical application such as it spreads easily, exhibiting maximum slip and drag. This nanosized formulations enhance permeability of drug extend retention at the site of action and the CMGF-ITZ-nPEVs gel shown greater antifungal activity than the marketed gel. The ITZ-nPEVs revealed here provide a novel therapy option for nail-related illnesses such onychomycosis. Clinical trials to determine the effectiveness of ITZ-nPEVs in patients with onychomycosis are presently underway.

## Figures and Tables

**Table 1:** Composition and characterization of ITZ-nPEVs Batches.

Batches	Component (% *w*/*v*)			Drug Content (mg/5 mL of nPEVs) *	% EE *	Particle Size (nm) *	PDI *	Zeta Potential (mV) *
	ITZ	Lipid	Cholesterol					
S1	1	4	-	95.36 ± 0.31	95.36 ± 0.51	196.55 ± 0.025	0.092 ± 0.03	+11.1 ± 0.52
S2	1	4	2	96.59 ± 0.44	96.59 ± 0.44	221.2 ± 0.056	0.19 ± 0.045	+17.2 ± 0.35
S3	**1**	**7**	**3**	98.43 ± 0.32	97.22 ± 0.46	240.33 ± 0.016	0.38 ± 0.01	+19.1 ± 0.87
S4	2	7	3	193.89 ± 0.8	96.94 ± 0.70	252.2 ±0.019	0.49 ± 0.011	+22.5 ± 0.28

* Each value represents mean, *n* = 3 ± SD.

**Table 2:** Deformability index and viscosity of ITZ-nPEVs and CMFG-ITZ-nPEVs formulations.

ITZ-nPEVs				CMFG-ITZ-nPEVs			
Sr. No.	Batches	Deformability Index (mL·s^−1^)	Viscosity (cP) *	Batches	CMFG (*w*/*v*)	Chitosan (*w*/*v*)	Viscosity (cP) *
1	S1	1.35	0.98 ± 0.02	G1	1%	1%	155.7 ± 0.567
2	S2	0.556	1.32 ± 0.015	G2	0.5%	1%	116.1 ± 0.85
3	S3	0.449	1.41 ± 0.032	G3	1%	0.5%	132 ± 0.737
4	S4	0.200	1.72 ± 0.025				

* Each value represents mean, *n* = 3 ± SD.

**Table 3:** In vitro release kinetics of ITZ with various nPEVs and CMFG-ITZ-PEVs (mean ± SD, *n* = 3).

Correlation Coefficient (R^2^)						
Formulation	Zero-Order	First Order	Higuchi	Hixon Crowell	Korsmeyer- Peppas	Release Exponent (*n*)
Pure ITZ	0.9612 ± 0.48	0.7433 ± 0.67	0.9715 ± 0.34	0.8920 ± 0.19	0.8912 ± 0.17	0.417 ± 0.35
S1	0.9766 ± 0.12	0.7466 ± 0.28	0.9726 ± 0.21	0.8707 ± 0.14	0.8865 ± 0.15	0.478 ± 0.38
S2	0.9874 ± 0.22	0.7762 ± 0.19	0.9614 ± 0.23	0.9101 ± 0.23	0.9244 ± 0.15	0.588 ± 0.18
S3	0.9895 ± 0.16	0.7492 ± 0.15	0.9788 ± 0.31	0.9189 ± 0.16	0.9779 ± 0.33	0.613 ± 0.43
S4	0.9924 ± 0.31	0.7756 ± 0.19	0.9732 ± 0.24	0.9134 ± 0.23	0.9584 ± 0.32	0.598 ± 0.31
CMFG-ITZ-PEVs	0.9912 ± 0.34	0.7652 ± 0.23	0.982 ± 0.16	0.9212 ± 0.31	0.9712 ± 0.25	0.591 ± 0.43

**Table 4:** Nail hydration study of various formulation.

Sr. No.	Groups	Formulations	Weight of Nails before Hydration (mg)	Weight of Nails after Hydration (mg)	HE24 (h)
**1**	Control (group I)	Deionized water	50	49.2	-
**2**	Formulation (group II)	S1	50	62.0	1.26
		S2	50	68.2	1.38
		S3	50	75.6	1.53
		S4	50	72.7	1.47
**3**	CMFG-ITZ-nPEVs (group III)	-	50	81.11	1.92
**3**	Marketed gel (group IV)	Itrostred gel	50	52.65	1.07

**Table 5:** Antifungal activity of various formulation.

Sr. No.	Formulations	Zone of Inhibition * (mm)
1	Control	5.1 ± 0.12
2	Optimized batch (S3) of nPEVs	27.0 ± 0.25
3	CMFG-ITZ-nPEVs gel	33.2 ± 0.11
4	Itrostred Gel	22.9 ± 0.44

* Each value represents mean, *n* = 3 ± SD.

**Table 6:** Stability study of CMFG-ITZ-nPEVs gel.

Parameters	0 Month	1 Month	3 Month	6 Month
Drug Content * (mg/ 5 mL of nPEVs)	98.43 ± 0.32	98.39 ± 0.25	98.35 ± 0.39	97.21 ± 0.12
% Entrapment Efficiency *	98.43 ± 0.32	98.39 ± 0.25	98.35 ± 0.39	98.21 ± 0.12
Particle size * (nm)	240.33 ± 0.016	241 ± 0.47	299.25 ± 0.29	349.33 ± 0.92
Zeta potential * (mV)	19.1	19.3	19.5	19.6

(* Each value represents mean, (*n* = 3) ± SD).

## Data Availability

Data sharing not applicable.

## Supplementary Materials

The following supporting information can be downloaded at: https://www.mdpi.com/article/10.3390/polym14020325/s1, Figure S1: (**A**) Quantification chromatogram of ITZ, and (**B**) standard calibration curve of ITZ; Figure S2. (**a**) PS and PDI, (**b**) ZP of S3; Table S1: Peak area of ITZ at different concentration.
