# Supplementary material for: The Unique Carboxymethyl Fenugreek Gum Gel Loaded Itraconazole Self-Emulsifying Nanovesicles for Topical Onychomycosis Treatment
Source: Polymers (Basel). 2022 Jan 14;14(2):325. doi: 10.3390/polym14020325 (PMC8779379; doi:10.3390/polym14020325)
Supplement: Supplementary file 1 [file polymers-14-00325-s001.zip › polymers-1537303-supplementary.pdf]

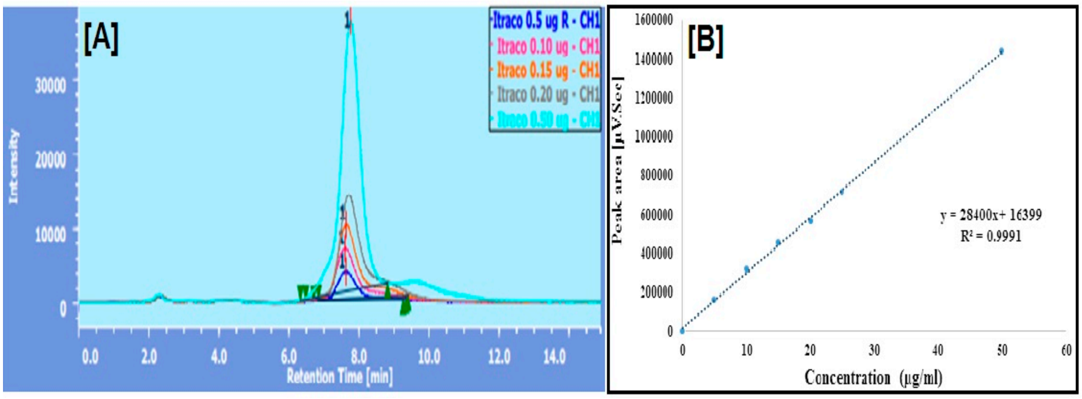

Figure S1. (**A**) Quantification chromatogram of ITZ, and (**B**) standard calibration curve of ITZ.

Table S1. Peak area of ITZ at different concentration.

Sr. No.	Concentration (µg/mL)	Peak area [µV.sec]
1	0	0
2	5	164355
3	10	320959
4	15	454341
5	20	569434
6	25	716822
7	50	1438885

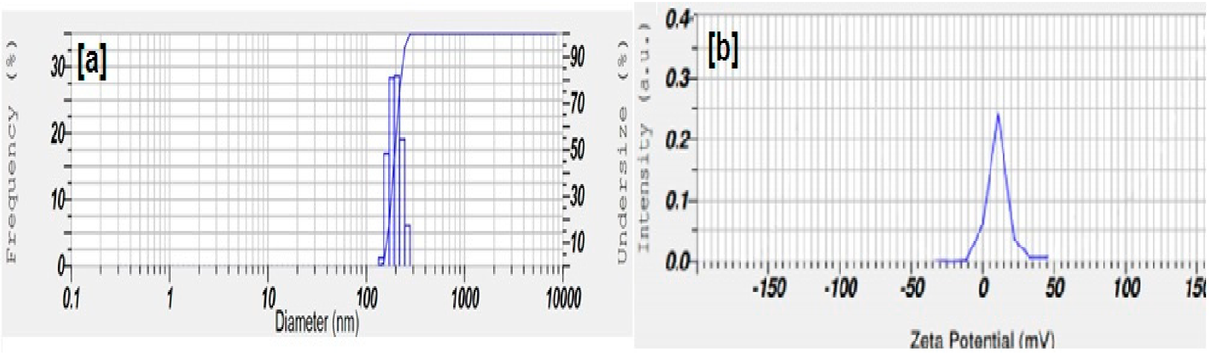

Figure S2. (**a**) PS and PDI, (**b**) ZP of S3.
